# The impact of postoperative pain interventions on circadian rhythm disruptions: mechanisms and clinical implications

**DOI:** 10.3389/fnins.2025.1543421

**Published:** 2025-04-28

**Authors:** Dongmei Ma, Li Li, Wei Liu, Jianhong Xu

**Affiliations:** ^1^Department of Anesthesiology, The Fourth Affiliated Hospital of School of Medicine, International School of Medicine, International Institutes of Medicine, Zhejiang University, Yiwu, China; ^2^Department of Orthopedics, The Fourth Affiliated Hospital of School of Medicine, International School of Medicine, International Institutes of Medicine, Zhejiang University, Yiwu, China

**Keywords:** pain, postoperative, pain management, circadian rhythm, sleep disorders, physiological process

## Abstract

Postoperative pain is a prevalent clinical issue that significantly impacts patient recovery, making its management crucial for rehabilitation. Recent studies have shown that postoperative pain not only affects the physiological state of patients but may also disrupt their circadian rhythms, leading to decreased sleep quality and physiological dysfunctions. This review aims to explore the effects of postoperative pain interventions on circadian rhythm disturbances, analyze the underlying mechanisms, and summarize the effective strategies currently used in clinical practice. Through a comprehensive analysis of the relevant literature, we will highlight the importance of pain management during the recovery process and emphasize its potential role in regulating circadian rhythms. Pharmacological treatments like NSAIDs and melatonin have shown efficacy in regulating circadian rhythms and improving sleep quality in postoperative patients. Multimodal analgesia combining pharmacological and non-pharmacological methods (e.g., CBT, acupuncture) can optimize pain relief while minimizing side effects. However, further research is needed to clarify the bidirectional relationship between pain perception and circadian rhythms and translate these findings into clinical practice.

## 1 Introduction

Postoperative pain represents a significant challenge for patients after surgery, greatly affecting their comfort and recovery process. Managing this pain effectively is crucial, as it not only enhances patient satisfaction but also contributes to better recovery outcomes. Recent studies have highlighted the intricate relationship between postoperative pain and circadian rhythms, suggesting that pain can disrupt the body’s natural biological clock, which in turn negatively affects sleep quality and various physiological functions (Zee et al., 2013; Guo et al., 2023; Jiang et al., 2024). This review aims to explore how interventions aimed at managing postoperative pain influence circadian rhythms, while also clarifying the underlying mechanisms and clinical implications of this relationship. After surgery, patients often experience changes in their sleep patterns and circadian rhythms, both of which are essential for maintaining homeostasis and overall health. Disruptions in these rhythms can worsen pain perception, creating a harmful cycle that hinders recovery (Barnadas Solé et al., 2021; Li et al., 2023; Šmon et al., 2023). This review aims to systematically evaluate how postoperative pain interventions modulate circadian rhythm disruptions, analyze underlying mechanisms, and summarize evidence-based strategies for clinical implementation.

A multi-center, prospective cohort study revealed that 53.9% of surgical patients experienced severe postoperative pain (Numerical Rating Scale ≥7) within the critical 0–24 h postoperative window. This nociceptive burden was significantly associated with impairments in mood, ambulation, deep breathing, and sleep quality, alongside increased incidence of vertigo, nausea, and fatigue (*p* < 0.05) (Emrich et al., 2023). Notably, this statistical pattern has shown no significant improvement across three decades of observational research, underscoring the urgent need for innovative analgesic strategies (Wu et al., 2024). For instance, research shows that patients with severe postoperative pain frequently report poor sleep quality, which can lead to increased pain sensitivity and longer recovery times (Büyükyilmaz et al., 2011; Wylde et al., 2011). The connection between pain and circadian rhythms is complex, involving various biological systems such as hormonal regulation, immune responses, and neurophysiological mechanisms (Santos et al., 2020; Bumgarner et al., 2021; Zhu et al., 2024). A thorough understanding of these interactions is vital for developing effective pain management strategies that not only aim to relieve pain but also support the restoration of circadian rhythms and improve overall health (Kaur and Shyu, 2018; Seiger et al., 2024).

The onset of chronic postoperative pain can result in a range of ongoing complications, often presenting as chronic pain syndromes that may be influenced by disruptions in circadian rhythms (Liu et al., 2021; Suarez-Roca et al., 2024). Consequently, it is essential for healthcare providers to implement a multimodal approach to pain management that takes into account the circadian factors affecting both pain perception and recovery (Al-Waeli et al., 2020; Tanaka et al., 2021; Daguet et al., 2022).

While previous research has touched on the relationship between postoperative pain and circadian rhythms, there is a lack of comprehensive analysis and practical guidance for clinicians. While previous studies have linked postoperative pain to circadian dysfunction, few reviews have synthesized the bidirectional relationship or translated findings into actionable clinical guidelines. This review aims to explore the various interventions available for managing postoperative pain, their impact on circadian rhythms, and the potential to enhance patient outcomes through focused pain management strategies.

### 1.1 Physiological mechanisms of postoperative pain

The physiological mechanisms underlying postoperative pain involve a complex interplay between the peripheral and central nervous systems in the transmission and processing of nociceptive signals. This process begins when peripheral nociceptors identify harmful stimuli, converting them into electrical impulses that travel along afferent nerve fibers to the spinal cord. Within the spinal cord, the relay of pain signals is facilitated by several receptors, including transient receptor potential (TRP) channels and purinergic receptors, which are vital in modulating pain perception (Khan et al., 2019; Yang L. et al., 2023). Upon reaching the dorsal horn of the spinal cord, these signals undergo additional processing, during which excitatory neurotransmitters such as glutamate and substance P are released, further amplifying the pain response (Han et al., 2021).

The phenomenon of central sensitization can occur when the spinal cord becomes more responsive to stimuli, leading to increased sensitivity to pain and the potential development of chronic pain (Lai et al., 2024). This is particularly relevant in postoperative situations, where surgical injuries can sensitize the nervous system, resulting in heightened pain experiences after surgery. Research has shown that hypersensitivity to pain before surgery can predict the severity of postoperative pain, highlighting the importance of assessing pain sensitivity prior to surgical procedures (Del Tedesco et al., 2023; Lai et al., 2024). Understanding how pain signals are transmitted and processed is crucial for developing effective pain management strategies in postoperative care.

The central nervous system (CNS) plays a key role in how pain is perceived and modulated, especially during recovery after surgery. Pain signals processed in the spinal cord are sent to various brain regions, including the thalamus and cortex, where emotional and cognitive aspects of pain are integrated. The brain not only interprets these signals but also modulates them through descending pathways that can either enhance or diminish pain perception (Zhang et al., 2023).

In the context of postoperative recovery, acute pain can trigger neuroinflammatory responses that heighten pain perception and may lead to cognitive deficits, especially in older adults (Zhao et al., 2021). This neuroinflammatory response is driven by the release of pro-inflammatory cytokines, which can sensitize neurons and change synaptic plasticity, resulting in persistent pain. Furthermore, neurotrophic factors like brain-derived neurotrophic factor (BDNF) are essential for pain modulation and recovery following surgical procedures (Rajamanickam et al., 2024). Understanding the CNS’s response to pain is crucial for developing targeted therapies that can mitigate postoperative pain and its associated complications, ultimately improving patient outcomes in surgical settings.

### 1.2 Biological basis of circadian rhythm

Circadian rhythms are innate biological mechanisms that function on a roughly 24 h cycle, playing a crucial role in regulating various physiological processes across different organisms (Ikonomov et al., 1998; Wang et al., 2021). At the heart of these rhythms is the circadian clock, primarily located in the suprachiasmatic nucleus (SCN) of the hypothalamus in mammals, which serves as the main pacemaker (Schwartz et al., 1980; Liang et al., 2025). This nucleus is essential for synchronizing peripheral oscillators found in nearly all tissues, ensuring that the organism’s physiological functions align with external environmental signals, especially the cycles of light and darkness. The molecular basis of the circadian clock involves a complex network of genes and proteins that interact in feedback loops to regulate the expression of clock-related genes. For instance, acute surgical injury triggers a cascade of pro-inflammatory cytokines, including tumor necrosis factor-*α* (TNF-α) and interleukin-6 (IL-6), which disrupt circadian clock gene expression in both the suprachiasmatic nucleus (SCN) and peripheral tissues (Paladino et al., 2014). These cytokines downregulate the transcription of core clock genes BMAL1 and CLOCK, while upregulating PER2 and CRY1 in a time-dependent manner, leading to desynchronization between the central and peripheral circadian oscillators (Guo et al., 2015). For instance, TNF-α-mediated inflammation directly impairs SCN function by altering glutamate receptor signaling, thereby blunting the SCN’s response to light–dark cues (Paladino et al., 2014). Recent studies have highlighted the importance of post-translational modifications in the stability and function of these clock proteins, emphasizing the dynamic nature of circadian regulation and its evolutionary conservation across various species (Philpott et al., 2022; Sharma and Partch, 2024).

Circadian rhythms significantly influence a range of physiological states, including metabolism, sleep–wake cycles, hormonal secretion, and immune responses (Cable et al., 2021; Liu et al., 2022). Disruptions to these rhythms, often seen in scenarios like shift work or chronic jet lag, can lead to negative health outcomes, such as metabolic disorders, cardiovascular problems, and mood disturbances. For example, research indicates that misalignment of circadian rhythms can negatively impact glucose metabolism and insulin secretion, thereby heightening the risk of developing type 2 diabetes (Ashraf et al., 2019). Circadian rhythms play a crucial role in regulating the gastrointestinal system, and when these rhythms are disrupted, it can lead to functional gastrointestinal disorders (Fowler et al., 2022). Research shows that these rhythms also have a significant impact on mental health, with disturbances exacerbating conditions like bipolar disorder and depression (Jermann et al., 2020). Additionally, the timing of medication intake, known as chronotherapy, is increasingly recognized as essential for enhancing therapeutic effectiveness, as the metabolism and effectiveness of drugs can be greatly influenced by the body’s circadian rhythms (Lassi et al., 2021). Understanding the biological underpinnings of circadian rhythms is therefore vital for developing strategies to mitigate the health consequences of circadian disruption and to promote overall well-being.

The PER3 VNTR polymorphism changes how pain is regulated throughout the day. Carriers of the PER3(5/5) genotype show reduced pain inhibition in the afternoon compared to those with the PER3(4/4) genotype. This reduction is linked to sharper decreases in serum BDNF and S100B levels during the day (Carvalho et al., 2019). This finding is consistent with data from myocardial infarction that indicate PER3(5/5) genotypes are linked to pain that occurs primarily in the morning, along with increased inflammatory markers (Lipkova et al., 2014).

### 1.3 Postoperative pain and circadian rhythm relationship

The bidirectional interaction between postoperative pain and circadian rhythms is increasingly recognized as a critical determinant of recovery outcomes. Postoperative pain is not just a physiological response to surgical injury; it also follows a circadian rhythm dictated by the body’s internal biological clock. Studies indicate that the severity of pain and inflammatory responses fluctuate throughout the day in line with these rhythms, which are regulated by clock genes (Rodríguez-Palma et al., 2025). For example, research has shown that administering non-steroidal anti-inflammatory drugs (NSAIDs) during the active phase of the circadian cycle can lead to better pain management and recovery compared to giving them during the inactive phase (Al-Waeli et al., 2020). This is because the body’s natural healing processes, including the release of anti-inflammatory cytokines, are more effective at specific times of the day. Therefore, pain management strategies should consider the timing of medication to align with these biological rhythms for optimal results (Al-Waeli et al., 2020; Tamimi et al., 2022).

Circadian rhythm disorders can manifest in various clinical symptoms, especially during surgical interventions. Patients may face sleep disturbances, heightened sensitivity to pain, and increased anxiety and delirium, all of which can impede recovery. For instance, studies have shown that surgeries performed at night can lead to poorer postoperative outcomes due to misalignment with circadian rhythms, adversely affecting sleep quality and raising the incidence of postoperative delirium (Cicekci et al., 2024; Jiang et al., 2024). Moreover, the impact of circadian rhythm disruptions on pain perception is significant, as certain pain conditions (Junker and Wirz, 2010; Knezevic et al., 2023), like fibromyalgia, exhibit specific fluctuations in pain intensity throughout the day (Korszun, 2000). Healthcare professionals should consider the timing of surgical procedures and pain relief methods to align with patients’ circadian rhythms, as this synchronization is essential for improving recovery outcomes and minimizing complications (Knezevic et al., 2023; McEachern et al., 2024). Effectively managing postoperative pain while taking circadian factors into account can significantly enhance patient experiences and overall recovery paths.

Melatonin disruption plays a critical role in the interplay between pain and circadian dysfunction. Chronic pain not only significantly disrupts circadian rhythms but also creates a harmful cycle that intensifies both pain and sleep-related issues (Chen et al., 2016; Kaur and Shyu, 2018; Fujimoto et al., 2024). Specifically, postoperative pain can inhibit the synthesis of melatonin by reducing the activity of arylalkylamine N-acetyltransferase (AANAT), the key enzyme in melatonin production. As melatonin levels drop, patients often experience poor sleep quality and heightened pain perception, given that melatonin has direct analgesic effects through the modulation of spinal cord glutamate release via MT1/MT2 receptors.

This relationship is further evidenced by the temporal variations in pain perception. Research indicates circadian-dependent fluctuations in nociceptive thresholds, with increased sensitivity noted in the late evening and decreased sensitivity in the early morning (Kubynin and Ignatov, 1996). Patients undergoing abdominal surgery, for instance, report significantly higher pain intensity at night, which aligns with diminished melatonin secretion and elevated pro-inflammatory cytokines such as IL-6. This chronic state of sleep fragmentation due to pain exacerbates the production of pro-inflammatory cytokines, perpetuating circadian misalignment.

**Figure 1:**
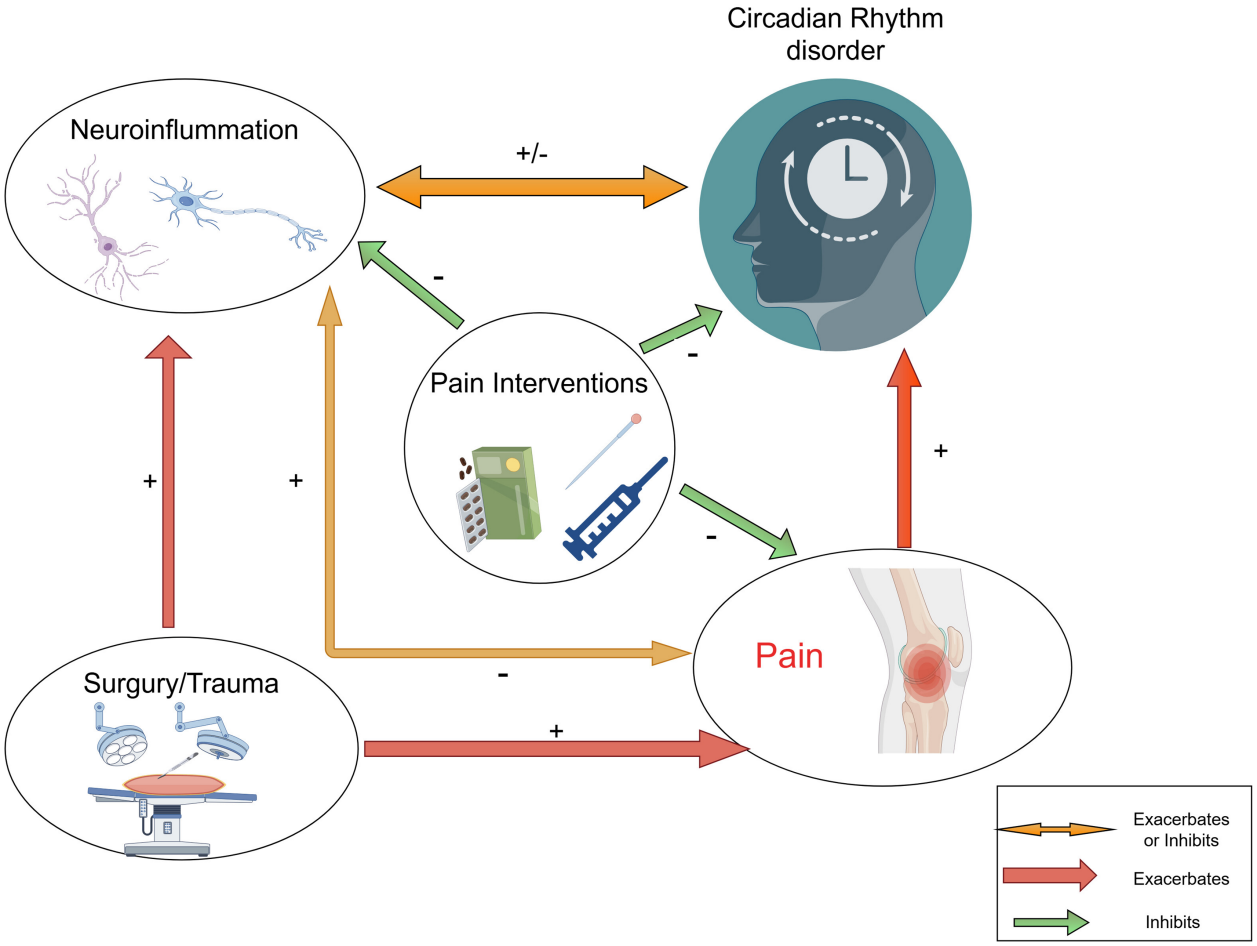
Interactions between circadian rhythms disorder, neuroinflammation and pain interventions. The figure illustrates the links between pain, neuroinflammation (including glial cells and neurons), and circadian rhythms. Further study is needed to understand the mechanisms regulating circadian pain.

Moreover, the body’s response to pain can disrupt hormonal balance, particularly affecting melatonin production, which is essential for maintaining circadian rhythms (Gögenur et al., 2007). The resulting sleep disturbances not only impede recovery but also elevate the risk of postoperative complications. Effective pain management is thus crucial to prevent disruptions in circadian rhythms and facilitate a more efficient recovery process (Tan et al., 2019; Al-Waeli et al., 2020). Chronic pain significantly disrupts circadian rhythms, creating a harmful cycle that exacerbates both pain and sleep-related issues. The body’s response to pain can interfere with hormonal balance, particularly affecting melatonin production, which is crucial for regulating circadian rhythms. When pain leads to sleep disturbances, it can impede recovery and increase the risk of postoperative complications. Research shows that patients experiencing acute postoperative pain often report sleep issues, which may worsen pain perception and elevate anxiety levels (Al-Waeli et al., 2020; Yang J. et al., 2023). This relationship highlights the importance of effective pain management to prevent disruptions in circadian rhythms, ultimately facilitating a more efficient recovery process (Vij et al., 2018; Yin et al., 2022). Additionally, understanding how pain affects circadian rhythms could lead to innovative treatments, such as using melatonin or other chronobiotic substances to mitigate these negative effects (Cicekci et al., 2024; Jiang et al., 2024) (Figure 1).

### 1.4 Pain intervention strategies and methods

Pharmacological treatments play a crucial role in pain management, providing a wide array of medications to address various pain types, including acute, chronic, and procedural discomfort. Recent advancements in pharmacotherapy have focused on refining existing medications and developing new agents that specifically target pain pathways. For instance, NSAIDs and opioids are commonly used, but their use is often tempered by concerns regarding side effects and the potential for addiction, especially with opioids (Boblewska and Dybowski, 2023). Consequently, there is a growing interest in alternative pharmacological options, such as innovative analgesics that interact with different receptors, including cannabinoids and certain antidepressants that have shown effectiveness in treating neuropathic pain (Hurley-Wallace et al., 2021). Additionally, the integration of pharmacogenetics into pain management strategies is starting to personalize treatment approaches, allowing for more tailored therapies based on individual genetic profiles that influence drug metabolism and effectiveness (Watanabe et al., 2024).

Recent investigations have highlighted the importance of multimodal analgesia, which combines pharmacological agents with non-pharmacological methods to optimize pain relief while minimizing side effects. For example, using NSAIDs together with acetaminophen or adding therapies like gabapentinoids has shown to provide better pain management than using a single treatment alone (Boblewska and Dybowski, 2023). Additionally, the trend of drug repurposing has gained traction, particularly during the COVID-19 pandemic, as existing medications are being explored for new roles in pain management (Khadka et al., 2020). In summary, as pharmacological strategies evolve, the focus remains on improving effectiveness, reducing adverse effects, and integrating these therapies into a comprehensive pain management approach.

Non-pharmacological approaches have gained widespread recognition in pain management as critical complements or alternatives to pharmacological therapies (Wei et al., 2022). These methods-including cognitive behavioral therapy (CBT), physical therapy, acupuncture, and mindfulness-based interventions-have demonstrated efficacy in alleviating pain and improving overall quality of life (Garland et al., 2019; Klausen et al., 2019; Yang et al., 2021). For instance, CBT has shown significant effectiveness in chronic pain management by addressing psychological aspects of pain, enhancing coping strategies, and reducing pain-related disability (Thorn and Kuhajda, 2006; Smith et al., 2019; Urits et al., 2019, p. 51). Similarly, physical therapies such as physiotherapy and exercise have been validated for pain relief, particularly in musculoskeletal disorders, where they improve functionality and decrease reliance on medications (Fortún-Rabadán et al., 2021; Hurley-Wallace et al., 2021).

These integrative approaches not only optimize pain management but also enhance patients’ overall quality of life. Acupuncture is a significant non-drug approach that has gained recognition for its ability to relieve pain. Systematic reviews indicate that acupuncture can significantly reduce pain intensity, particularly in conditions like osteoarthritis and chronic lower back pain (Brinkhaus et al., 2003; Xu et al., 2013; Chang et al., 2022). Mechanistically, acupuncture modulates the hypothalamic–pituitary–adrenal axis, reducing cortisol levels and promoting melatonin synthesis (Li et al., 2021). Additionally, integrative methods that combine various non-drug techniques, such as mindfulness meditation and yoga, have shown promise in relieving pain and enhancing psychological well-being. This suggests that a comprehensive approach to pain management could yield the best results (Wolkin, 2015; Zeidan et al., 2015; Johnston et al., 2023).

**Figure 2:**
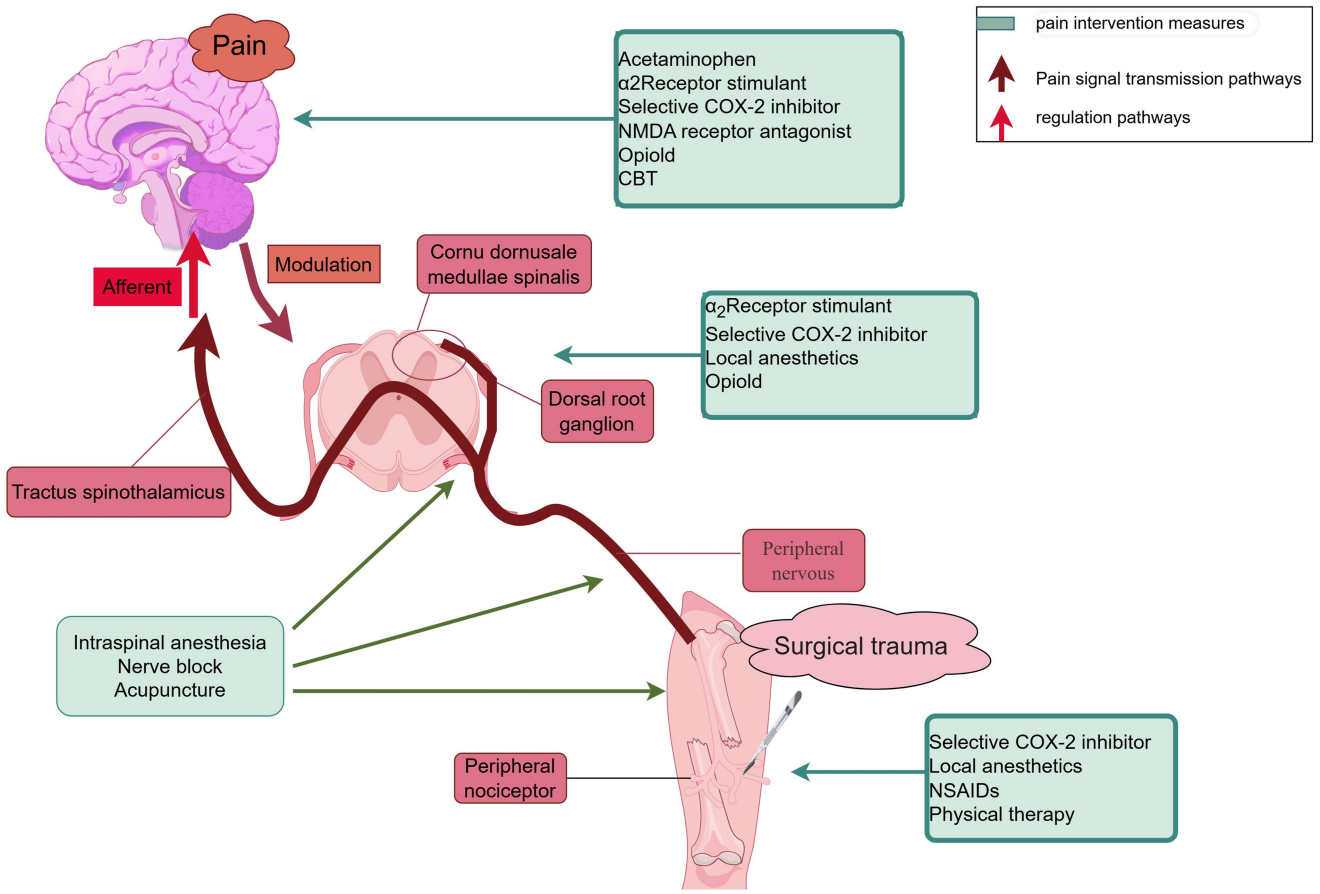
Schematic diagram of pain management and drug mechanism of action. The figure illustrates the basic principles and interrelationships of different pain management methods, helping to understand how to improve pain management effects through these interventions. NSAIDs, non-steroidal anti-inflammatory drugs; CBT, cognitive-behavioral therapy.

Postoperative pain management involves a variety of techniques targeting different mechanisms within the pain pathway, ranging from local anesthetics to systemic medications like opioids and NSAIDs. These strategies aim to alleviate pain by either blocking pain signals at their origin, reducing inflammation, or modulating the central nervous system’s perception of pain. An overview of the schematic diagram of pain management and drug mechanism of action, is depicted in Figure 2.

## 2 Potential areas and challenges for future research

Future research should focus on areas like personalized medicine, digital health monitoring, and the discovery of new biomarkers. The increasing use of smartwatches and other digital health technologies calls for effective integration of these tools into pain management, representing a key area for further exploration (Xie et al., 2021; Imeraj et al., 2022). However, future research must tackle challenges such as addressing ethical issues in current clinical trials, variability in patient selection, and barriers to data sharing (Morain et al., 2022; Møller et al., 2024). Progress in these areas will advance medical research, improve clinical treatment outcomes, and ultimately enhance patients’ quality of life.

The circadian rhythms play a crucial role in enhancing patient comfort and optimizing recovery outcomes. This systematic review emphasizes the increasing recognition of effective pain management strategies as essential for postoperative recovery and overall quality of life. The interplay between pain and circadian rhythms is complex, influenced by biological, psychological, and social factors, necessitating a multifaceted approach to pain management that considers these diverse perspectives. While traditional analgesic methods have been thoroughly examined, there is an urgent need to explore innovative strategies, including multimodal analgesia and non-pharmacological interventions, to assess their effectiveness and potential integration into clinical settings. Future research should focus on the bidirectional relationship between pain perception and circadian rhythms, investigating how disruptions in circadian cycles can exacerbate pain and vice versa. Gaining insights into these interactions could lead to the creation of tailored pain management strategies that align with patients’ biological rhythms, ultimately enhancing recovery outcomes. Additionally, fostering interdisciplinary collaboration among healthcare providers, researchers, and patients is crucial for advancing this field. Engaging patients in their pain management choices can empower them and potentially lead to better treatment outcomes.

Evidence highlights a two-way relationship between postsurgical pain and disruptions in circadian rhythms, which are influenced by neuroinflammatory pathways and neuroendocrine imbalances. To improve treatment outcomes, clinicians should consider timing analgesic medications in accordance with the body’s natural circadian rhythms. This involves scheduling nonsteroidal anti-inflammatory drug (NSAID) doses to coincide with periods of heightened pro-inflammatory cytokine activity, as determined by circadian biomarker profiling. Such a chronotherapeutic strategy could enhance the effectiveness of pain relief while reducing the risk of developing tolerance by aligning treatment with biological rhythms. Current guidelines suggest using a multimodal approach that combines *α*₂-adrenergic agonists with complementary therapies, such as electroacupuncture, to effectively manage pain signaling and correct circadian misalignment. Melatonin, which plays dual roles in pain relief and circadian regulation, can help reduce postoperative pain and restore normal sleep–wake cycles, particularly in patients with substantial circadian misalignment. A thorough assessment of circadian rhythms should include both subjective evaluations, like validated sleep questionnaires, and objective measurements, such as actigraphy to track rest-activity cycles, to accurately gauge circadian health.
